# Rose Wine Market: Anything but Colour?

**DOI:** 10.3390/foods9121850

**Published:** 2020-12-11

**Authors:** Stephanie Peres, Eric Giraud-Heraud, Anne-Sophie Masure, Sophie Tempere

**Affiliations:** 1 Groupement de Recherche en Économie Théorique et Appliquée (GREThA), Joint Research Unit CNRS, Institut des Sciences de la Vigne et du Vin (ISVV), University of Bordeaux, 33076 Bordeaux, France; 2 Groupement de Recherche en Économie Théorique et Appliquée (GREThA), Joint Research Unit CNRS, INRAE, Institut des Sciences de la Vigne et du Vin (ISVV), University of Bordeaux, 33076 Bordeaux, France; eric.giraud-heraud@u-bordeaux.fr; 3 Groupement de Recherche en Économie Théorique et Appliquée (GREThA), Joint Research Unit CNRS, University of Bordeaux, 33076 Bordeaux, France; anne-sophie.masure@u-bordeaux.fr; 4 Enology Research Unit, EA 4577, USC 1366, INRAE, Institut des Sciences de la Vigne et du Vin (ISVV), University of Bordeaux, 33076 Bordeaux, France; sophie.tempere@u-bordeaux.fr

**Keywords:** rose wine, sensory analysis, colour, experimental market, no added sulphites

## Abstract

In many countries, the consumption of still wine is in strong decline. The market for rose wine, however, stands in stark contrast to this trend, seeing worldwide growth of almost 30% over the last 15 years. For most observers/experts, product colour plays an important role in this paradigm shift. For this reason, companies’ marketing efforts often focus on this purely visual characteristic. There is, however, no certainty that other emerging consumer demands, related to environmental concerns or how “natural” a wine is (organic wines, natural wines, etc.), do not also play a role in the enthusiasm seen in new wine consumers. This article proposes an assessment of expectations related to colour and the decisions made by rose wine consumers, using two complementary experiments carried out in France. The first experiment is based on an online survey studying only consumers’ colour preferences. We will show that, contrary to popular belief, there is no consensus on this criterion, although regional trends can be identified. Typically, the “salmon” shade, which is generally the leader on the global market—and characteristic of Provence wines—does not win unanimous support across all regions. In contrast, an “apricot” shade seems to be preferred by consumers in the Bordeaux region. The second experiment confirms this result within the framework of an experimental market revealing consumers’ willingness to pay (WTP). This market also offers consumers the opportunity to taste wines and provides information on organic certification and “naturalness” (symbolised by the absence of added sulphites). We will then demonstrate how the latter criteria, although often popular, play only a small role—compared with colour—in consumer decisions. We will conclude this article with observations on the atypical nature of the rose wine market and on possible avenues for further research related to the emotional role colour plays in wine tasting and its possible specificity in the world of food and drink products.

## 1. Introduction

While the consumption of still wines has stagnated since the beginning of the millennium and has been in strong decline in most European countries, the rose market has continued to grow. Representing over 10% of the global consumption of still wines, worldwide consumption of rose wine has witnessed strong growth over the last 15 years, with a 28% rise between 2002 and 2017, culminating in sales of 25.6 million hectolitres in 2018 [1] (note that [1] refers to the worldwide consumption of rose wines that assumes a significant increase of 28% between 2002 and 2017, and reached 25.6 million hectoliters in 2018). France has tripled its consumption over the past 25 years, becoming the world’s leading consumer, with over 8 million hectolitres, representing approximately 35% of global consumption. In 2015, rose wine represented 31.2% of all wines consumed in France, versus only 10.8% in 1990 [2], with rose consumption in the country now overtaking the consumption of white wines [3]. Behind France, the United States alone absorbed 16% of the volume consumed worldwide in 2017 (with 5 million hl), far exceeding other markets (1.6 million hl for Germany, 1.2 million hl for the United Kingdom and 1 million hl for Italy) and in a climate of sustained growth (40% rise in imports of French rose wine in 2017). In these circumstances, exports of Provencal wines (the world’s leading regional producer, representing over 40% of the French market) have developed by some way, with growth of almost 300% over the last decade (31 million bottles exported in 2015 versus 8 million bottles in 2005). The export success of Provence rose wines is measured by record results, both in volume (+547% between 2008 and 2017) and value (+1020% over the same period), reaching EUR 226.2 million in 2017 according to the Conseil Interprofessionnel des Vins de Provence (data based on the French Customs statistics). These exports grow all over the world; nine countries imported more than 10,000 hl in 2017: the USA, UK, Belgium, Germany, Sweden, Netherlands, Switzerland, Australia, Canada.

The reasons for this enthusiasm are clearly linked to a paradigm shift in the consumption of wine. As certain marketing experts have emphasised, rose’s image is above all associated with celebration and pleasure: “Modern and transgressive, it breaks the codes of a universe reserved only for connoisseurs... Its pale colour is pleasing and gives the impression that it contains less alcohol than red wine...” (e.g., J.O Pesme (2018) [4]). Additionally, “fruity (not sweet), dry, low in alcohol... a wine that is easy to understand (and then drink); to sum up, a high-quality, sophisticated wine. From this observation, millennials aspiring to an opulent, epicurean lifestyle have adopted this product, identifying it as an accessible symbol of luxury that responds to their desire to drink something different from their parents.” [5]. New consumer habits thus reveal, through this specific market, a new relationship with wine (an opinion also shown by the Opinion Way survey for Wine Paris; [6]). Additionally, as so well shown in [7], the colour pink is indeed a driving force in this paradigm shift, since “the colour pink is an emotion” and “the colour of a certain form of self-expression”, so much so that rose wine surfs on this transgressive wave which has now infiltrated fashion, industrial brands, design, cosmetics, rugby team shirts and even politics. In marketing, the appeal of colour has been the subject of numerous studies and the psychology of colour is often highlighted [8,9]. Whether consciously or not, colour affects consumer perception and behaviour, confirming the reinforced emotional dimension of this characteristic [10,11]. In the case of rose wines, some analyses also attribute a reassuring, even soothing character to the colour [12].

It is therefore unanimously agreed that colour is a fundamental dimension for explaining the success of rose wine in markets and an important criterion in consumers’ purchasing decisions (see also [13,14,15]). Nonetheless, although rose wine delights millennials across the world by breaking with traditional codes (argument of [16], for whom the colour pink is also a “value”), it is also true that, in terms of wine, the process has a history. The Phocaeans first brought the production of “clear wine” to Provence in antiquity ([17,18]). In the Middle Ages, Bordeaux transformed it into “Clairet” for export to England, where it became the most popular wine in Britain until the 19th century. We can see then that this famous pink colour is in reality largely differentiated, both by its shade and its intensity [19], which ranges from pale “salmon-grey” for Provencal roses to dark “garnet-pink with deep purple highlights” for the Bordeaux Clairet (see the colour chart from the Centre du Rose in France: https://centredurose.fr/) [20]. Consequently—and rather unusually for the wine market—there is a desire to offer comprehensive distinctions for this single organoleptic characteristic. It is therefore possible to create an impressive chromatic identity map indicative both of the heterogeneity of consumer expectations and local production methods [14], despite a very clear evolution being observed over recent years [21]. Outside the European Union and the United States, the unusual cases of Uruguay (where rosé wine represents over 45% of the national wine consumption) and Tunisia (35%) are examples of atypical profile markets, with specific products in Uruguay (a fairly dark rosé) and a market led by tourist consumption in Tunisia. Uruguay (310,000 hl of rosé in 2017) is the world’s second largest consumer of rose per capita per annum. When it comes to the wine industry, this is highlighted in the example of the renowned brand “MATEUS”, created in Portugal in 1945 and exported in particular to the United States (with an annual production of over 50 million bottles, it is the fourth largest brand in the foreign wine market) [22]. It is worth noting that the colour of this wine is still positioned as a garnet shade, “recalling the colour of a Clairet”, that is radically different from Provencal rose.

Taking the viewpoint of consumers in the French market, this article shows the heterogeneity of expectations related to the colour of rose wine and the fundamentally decisive nature of this single organoleptic character. To do this, we conducted a preliminary online investigation, which suggests in particular that the salmon shade—generally the leader on the global market and typical of Provencal wines—does not in fact win unanimous support across all regions. The results of this survey were then confirmed in an experimental market (a similar experiment to the one proposed in [23,24] in terms of methodology) limited to consumers in the Bordeaux region. We can observe a certain consistency between our two experiments, insofar as consumers in the Bordeaux region systematically favour an “apricot” shade. The experimental market, however, enabled us to carry out a more in-depth analysis of behaviour. The aim was to ask consumers their willingness to pay for different types of wines, selected in advance, whilst simultaneously measuring the effects of other organoleptic characteristics, as well as information on sustainable production methods, particularly those related to “naturalness” (through the reduction of added sulphites) and environmental certifications (via the Organic Agriculture logo). We will show how the latter criteria have little impact on consumer choices when compared with colour. We will conclude this article on the atypical nature of the rose wine market and goals for research on the potentially emotional impact that colour may have on wine tasting.

From a sensory perspective, the most interesting arguments found in the literature studying the influence of wine colour on assessment are based on the possible link between this visual anchor and odour, aroma and taste evaluations. Colour (defined by the shade and intensity) does not therefore seem to be a characteristic that needs to be assessed independently from other characteristics, or, rather, other characteristics cannot be assessed independently from colour. Visual observation creates a phenomenon of expectation linked to aromas [25] which, psychologically speaking (e.g., [26]), can be interpreted as an integration of the context into the subject perceived, or, more specifically, into its representation, with the mind expecting to perceive specific aromas based on the colour observed. The visual therefore has a priming effect on the olfactory senses. This means that colour contributes to the anticipation of the odours, aromas and tastes of a wine, therefore subjectively guiding the overall assessment of a wine [27]. Consequently, significant changes in the descriptions of wines have been observed after artificially modifying the shade of a white wine using anthocyanins [28]. A white wine that has been “artificially modified” to look like a red wine after colouration therefore receives olfactory remarks containing vocabulary most commonly associated with red wine. The same concept has been found in early works [29]. By changing the shade of a white wine to make it look like a rose wine, these authors have already observed an increase in the perception of sweetness amongst tasters.

These results must nonetheless be accompanied by a wide range of reading, which, although less focused on colour, aims to assess the importance of all intrinsic wine characteristics (i.e., those which cannot be changed without modifying the product) in consumer choices [30,31]. The intrinsic characteristics of a product are not, however, sufficient for understanding consumer expectations, and market demand is also measured by reactions to extrinsic characteristics (i.e., those which can be changed independently from the product). The brand and labelling [32], price, origin [33] and alcohol content—which is not always identifiable from the taste—can all influence the evaluation (see also [34,35]).

Faced with new social challenges, however, the wine industry has had to adapt to market demands in terms of concerns related to health and the environment, which advocate the ongoing quest for more “natural” food and drink products. First of all, the purely environmental issue is attracting a growing number of consumers and it has been possible to measure how these consumers desire information/claims moving towards a reduction in the use of pesticides [23,36,37] and value organic wine certifications [38,39,40]. Next, the question of “naturalness” concerns objectively measurable aspects, such as a reduction in added sulphites used in the production of wines. The studies [41,42] measured these specific effects on wine. Reference [41] quotes the work of Amato, Ballco, Lopez-Galan, DeMagistris and Verneau (2017). These authors studied the market for wines without added sulphites in Italy and Spain. Reference [42] takes the results of Peyronnet’s 2018 paper, which assesses the impact of the statements with or without sulphites on French consumers. These two articles are complementary as they show that consumers in these three countries are ready to pay a significant additional price for a wine without added sulphites instead of a conventional wine. Looking at the market for wines with no added sulphites in Italy and Spain in the first instance, and then in France, they show that consumers in these three countries are willing to pay a significantly higher price for a wine with no added sulphites than they would for a conventional wine. These results also show a new aspiration among certain consumers, which could have a significant impact on the development of the wine market. To our knowledge, however, no study exists on consumers’ choices between the issue of “naturalness” and the organoleptic characteristics of rose wines.

## 2. Materials and Methods

### 2.1. Experiment 1

This first experiment aims to reveal the preferences of French consumers for rose wine in order to verify the predominance of an attraction to a specific colour.

An online survey on the perception of the “ideal colour” of rose wines among consumers was completed by 450 French consumers of wine. The survey questionnaire targeted men and women over 20 years old, living in one of these following three French regions: Ile-de-France, Gironde, Bouches-du-Rhone. To correspond to our target, the respondent had to consume red, rose or white wine on a regular or occasional basis.

Regarding the colour perception of the offered rose wines, a photo (in high definition) of rose wines bottles was displayed to the respondents, they were asked to rate the colour of the wine in each bottle, according to their own tastes. The picture of the entire range of rose wines (eight wines) was included in the online survey questionnaire. The order of the eight wines in this image was different from one respondent to another, with a random rotation set up every 50 respondents. Indeed, for each geographical area (Ile-de-France, Bouches-du-Rhone, Gironde), groups of 50 people were formed and each of these sub-groups received a photo of the eight bottles in a different order.

Respondents were informed at the start of the questionnaire that the survey was for a research project related to rose wine, and that their data would be processed confidentially and only within the framework of the study.

We chose to focus on three departments—representing major regions of France—in order to gain an in-depth understanding of the different consumer trends across various regions in France:

- Ile-de-France (150 consumers)
- Bouches-du-Rhone (150 consumers)
- Gironde (150 consumers)

These three distinctions were made taking into account the following criteria:

- For the Provence region, rose wine represents 89% of total wine production [2]. We therefore considered the Bouches-du-Rhone department for this region;
- In Gironde—another of France’s major wine-producing regions— however, rose wine represents only 4% of the total production of Bordeaux wine [43];
- Ile-de-France was chosen because Paris is the third most popular city in the world for millennials, according to the Global Cities Index produced by the American consulting firm AT Kearney. Millennials are seen as the new consumers of rose wine.

#### 2.1.1. Samples

**Figure 1:**
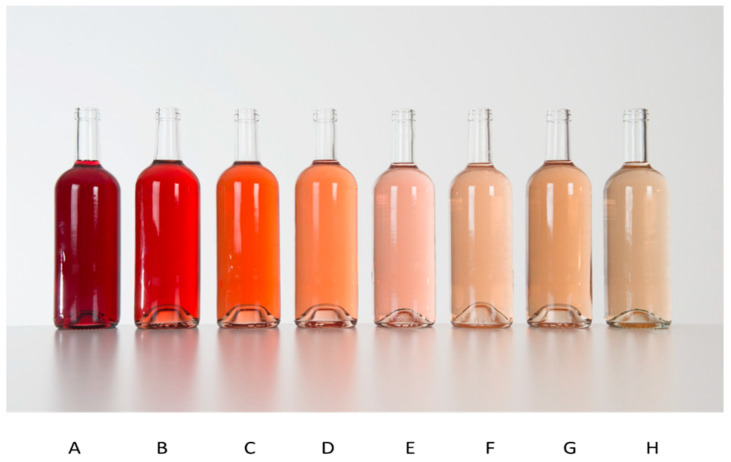
Photos of the eight wines selected and anonymised, representing a wide range of shades for rose wines on the market, based on the Colour Chart from the Centre du Rose (^®^) [44]. A–H: 8 shades of rosé wines (from the “garnet” shade to the “lychee” shade).

Eight wines were chosen by a group of experts based on their colour (the aim was to represent a wide range of wine shades, based on the Colour Chart for Rose Wines produced by the Centre du Rose). These wines were presented in neutral, transparent, Bordeaux-style bottles in order to ensure their anonymity (Figure 1). The order of the bottles was changed to eliminate any biases in subjects’ answers. Random rotations were completed every 50 surveys.

The sample of wines ranged from the “garnet” shade for Wine A to the “lychee” shade for Wine H, via the “apricot” shade for Wine D and “salmon” for Wine E. These terms, describing the colours observed, were at no point shared with the consumers.

All the wines presented to online survey respondents are commercial wines. However, so that consumers’ judgment was based exclusively on the colour of these wines, they were presented anonymously and in standardised bottles. Therefore, we did not indicate the different rose wines we chose in the paper.

#### 2.1.2. The Surveys

The survey questionnaire was designed in such a way as to collect information about the following general characteristics:

- Respondent profile (age, gender, socio-professional category, region);
- Information on wine consumption (rose wine, all wine colours, etc.);
- Information about purchasing practices and occasions on which rose wine is consumed;
- Consumption and purchase frequency;
- Period of consumption (summer, winter, throughout the year);
- Perception of colour (using a photo and value scale);
- Anticipation of aromas based solely on colour (from a list of aroma descriptors).

The following table (Table 1) represents the structure of the panel of respondents based on these different characteristics.

#### 2.1.3. Data Processing and Assessment Grid

To assess colour preferences for rose wines, we asked each consumer to associate each wine presented with a verbal statement relating to their visual assessment. A scale composed of six verbal categories was proposed: “Parfait” (perfect), “Tres bien” (very good), “Assez bien” (fairly good), “Moyenne” (average), “Pas terrible” (not great), “A rejeter” (rejected). This assessment was made through comparison (consumers could observe all eight wines at the same time).

To aggregate preferences, we used the “majority judgement” system originally proposed by Balinski and Laraki [45] and applied to wine classifications by the same authors [46]. This methodology, the adequacy of which is discussed by the authors, led to the selection of the statement which would be accepted by more than 50% of respondents (supposing that a respondent choosing a more complimentary statement than the majority statement would also accept the majority statement).

### 2.2. Experiment 2

The aim of the experimental market was to ask consumers their willingness to pay for different types of wines, whilst measuring, on the one hand, the role of visual anchoring, and, on the other hand, the effects of other organoleptic characteristics, as well as information related to different production methods.

#### 2.2.1. The Wines

For this second experimental economics study of consumers using an incentive-aligned procedure, colour differences, certifications and claims were the primary criteria in the selection of wines. Three wines with different shades and production methods were chosen from the same producer. The selection of wines by shade was once again carried out by a group of professionals using the colour chart for rose wine.

The wines in question were commercial wines from the southwest region of France (Buzet appellation) from the 2016 vintage.

Table 2 summarises the main characteristics of each wine.

These measurements were made by the wine company who provided them to us together with the wines’ technical descriptions.

The first wine used for the experiment was a wine with no added sulphites, produced from 100% Merlot, characterised by the “apricot” shade according to the Centre du Rose colour chart (corresponding to the shade of Wine D in Experiment 1). The second wine in the experiment was a conventional wine, characterised by the “salmon” shade in the colour chart (corresponding to the shade of Wine E in Experiment 1). The third wine introduced was an organic wine, characterised by the “lychee” shade (corresponding to the shade of Wine H in Experiment 1). These last two wines were blends.

The organic wine was used twice in the experiment. Its colour was modified using food colouring (Vahine^®^, red: azorubine, blue: Brilliant Blue FCF, yellow: tartrazine) so that it resembled a wine with no added sulphites.

A first triangular test (ISO 4120: 2007) in transparent INAO glasses (ISO 3591: 1977) was completed with 31 subjects (each subject completed the test twice). In this case, the evaluation was purely visual and compared the modified organic wine with the wine with no added sulphites. This test made it possible to significantly validate non-differentiation between the two samples (1/3 binomial probability law; 24 correct answers out of 62 tests; *p*-value = 0.22).

A second triangular test in black INAO glasses was also completed by 24 subjects (without repetition) and compared the modified wine with the organic wine (retro-nasal evaluation). The samples were compared by taste and aromas. The results show that there was no significant difference between the two samples (1/3 binomial probability law; nine correct answers out of 24 tests; *p*-value = 0.41). Consequently, the addition of colouring agents had no olfactory or gustatory impact on the wine.

#### 2.2.2. The Consumers

The participants, all inhabitants of the Bordeaux region, were recruited by phone by a company specialising in consumer studies. Various selection criteria were used:

- consumption of rose wine at least three times per year.
- purchase of rose wine at least twice per year.

From a more general perspective:

- subjects not in employment were limited to a maximum of 20%. Pensioners were not considered to be subjects not in employment.
- wine professionals were excluded from the study.
- the consumers as a whole represented two age groups (20–35 years; 55 years and over) and were equally distributed in terms of gender (50% women/50% men). These two generations were chosen based on literature relating to the question of generation in the consumption of wine [47,48,49]. The age brackets correspond to the baby boomer generation (traditional approach to wine in selection criteria) and generation “Y” or “millennials” (less frequent consumption, led by simplicity and variety).
- it was the first time that the consumers had participated in a sensory analysis session related to wine.

In total, 106 subjects took part in the study, half of whom were women. The average age was 48.5 ± 19 (min.: 20 years, max.: 81 years). The distribution of the age ranges targeted corresponded to 47 subjects of 20–35 years, and 59 subjects of 56 years and above. The average income for the entire panel was between EUR 1000 and EUR 1800 per month.

The consumers were registered at random for four separate sessions, with each session bringing together 26–28 people in June 2017. They were each paid EUR 30 by cheque for participating in the 2 h session (cheque provided at the end of the session). During the recruitment phase, the consumers were invited to fill in a questionnaire on their socio-demographic characteristics.

Before the start of the experiment, all participants provided informed, written consent, in particular, indicating the availability of spittoons and breathalysers. Additionally, consumers were told that they could stop the experiment at any time and that the information provided would be used only for scientific purposes and processed anonymously.

#### 2.2.3. Experiment Procedure

Sessions were completed in a sensory analysis room (ISO 8589: 2007) and each taster was placed in an individual booth. Twenty-five millilitres of each wine were presented in a transparent INAO glass (ISO 3591: 1977). The samples were presented anonymously (random three-digit coding) and served in a random order for each consumer. All sessions were held in the morning (10 a.m.) and tastings began at 11 a.m., following explanations given by the conductor of the experiment. Before beginning the experimental tasting, consumers therefore received instructions and a precise description of the willingness to pay procedure. The surplus method, which was tried and tested by [50], was chosen for direct revelation of consumers’ willingness to pay, in which consumers set a minimum purchase price, following comparison of the products proposed for sale. The wine sold to them would therefore be the wine that maximised their surplus (difference between the willingness to pay (WTP) and the sales prices drawn at random using the same original method proposed by [51]). The surplus method has been used recently in experimental economics research works [52].

Participants were asked not to communicate with one another during the experiment and were informed that they were allowed to drink water and eat bread after tasting each sample.

The experimental procedure was divided into four individual stages:

- Stage 1: “Anchoring stage”: Information on the name of the appellation and the vintage;
- Stage 2: “Sensory stage”: Evaluation of the organoleptic characteristics of wines;
- Stage 3: “Sulphites stage”: Stage providing regulatory information;
- Stage 4: “Label stage”: Stage providing information on the existence of organic agriculture (AB) certification.

The first stage was designed to provide consumers with minimum common references: only the name of the appellation and the vintage. This stage was designed to prevent cognitive interference and inter-individual differences linked to blind tasting. The authors of [53] reported that in the absence of a moral anchor, subjects deviate from their natural behaviour. They tend not to apply too much cognitive effort to the task and not to reveal their true preferences.

During the second stage, the consumers were invited to taste the wines, without restriction, following as naturally as possible the steps found in a classic tasting. They needed to taste the wines to assess all of their organoleptic characteristics. This second stage therefore positioned consumers in a re-purchasing act.

The procedure was therefore designed in this context of re-purchasing: having gained knowledge of the different wines after tasting them, the extrinsic characteristics were then revealed. The aim was to find out to what extent health and environmental considerations led to a reassessment of organoleptic characteristics.

The third stage informed consumers of health regulations. They learnt that some wines contained added sulphites, whilst others contained “no added sulphites”. Finally, the last stage provided environmental information on the type of grape growing used. Here, the consumers learnt that some wines were wines produced using organic agriculture (in which case the AB (organic agriculture) logo was featured on the label) and others were not.

At each stage, the consumers were invited to indicate their willingness to pay (WTP).

#### 2.2.4. Data Processing

If a consumer indicated a WTP of zero for all wines presented (complete rejection of the wines and the experiment), their results were not taken into consideration in the analysis of results. Thanks to our selection criteria, however, this did not occur.

The data were analysed using XLSTAT (2019.1.3) software (Addinsoft, Microsoft Excel, Paris, France). Three-way ANOVA (subject, wines and stages as independent variables with interaction) with post hoc Fisher Least Significant Difference (LSD) tests were applied to consumer data. However, when the conditions of the application of the ANOVA were not observed, a Friedman test was applied.

In order to verify the consensus or the heterogeneity of preferences, a principal component analysis (PCA) was applied with the tasters’ judgments as variables.

## 3. Results

### 3.1. Results from Experiment 1

The overall results of the survey are presented in Table 2.

In Table 3, for each wine presented to consumers, we have provided the colour reference from the Centre du Rose colour chart. The table shows the percentage obtained for each verbal statement.

With the “Assez bien” majority statement, Wine D is, in this sample, the preferred colour for the consumers in our panel, for all regions of France as a whole (Table 2); the colour of Wine D corresponds to the “apricot” shade in the Centre du Rose colour chart. Wine D is ahead of Wine E (wine in the “salmon” shade) but is, most importantly, ahead of the two extremes (Wine A: “garnet” shade, and Wine H: “lychee” shade). It should also be noted that Wine D has the fewest negative statements (with only 4% rejections), while it has 21% of votes for its “perfect” colour.

The majority statement is the statement chosen by a majority over all other statements. The majority statement for Wine D is “Assez bien” (fairly good), as a majority of 71% of consumers deemed the colour of this wine to merit at least the statement “Assez bien” (meaning that all lesser statements are supported only by a minority) and a majority of 55% of consumers deem it to merit at least the statement “Assez bien” (meaning that all more favourable statements are supported by a minority).

Nonetheless, this table masks a strong relation between results related to the geographical criterion. Table 3 describes in more detail, and by geographical area, results for respondent preferences for the colour of rose wines.

Thus, although respondents from Bouches-du-Rhone have a marked preference for the “salmon” shade of Wine F (60% of the “Tres bien” (very good) statements were attributed to this form), those from the Gironde and Ile-de-France regions voted primarily in favour of the shade of Wine D (“apricot” shade), with respective scores of 71% and 72% for the “Assez bien” statement (Table 4A–C).

In Ile-de-France, the wines are graded relatively positively, with the exception of the two extremes (wines A and H), which were both rejected by over a quarter of the population surveyed. This first table (Table 4A) shows that Bottle D, with the “apricot” shade, is the bottle with the most positive statements; consumers give it the majority statement “Assez bien” (fairly good), and it was rated by over a quarter as “parfaite” (perfect) and rejected by less than 5%.

Very similar results were found in Gironde (see Table 4B).

The majority judgement also shows that, in this region, Bottle D, with the “apricot” shade, is the bottle with the most positive statements, and that it has the fewest negative statements, as it is rejected in only 4% of cases (versus 33% for Bottle H, with the “lychee” shade, the lightest shade of all bottles surveyed).

For the Provence region, in the Bouches-du-Rhone department, the results are somewhat different. Bottle E, representing the “salmon” shade in the Centre du Rose colour chart, with the majority statement “Tres bien” (very good), is ahead of Bottle D, with the statement “Assez bien” (fairly good), even though the latter is the bottle with the fewest negative statements (only 4%). The majority judgement shows, however, that the pronounced colours of Bottle A and Bottle B (“garnet” and “cherry” shades, respectively, in the Centre du Rose colour chart) are rejected by 45% and 21% of consumers in this region (see Table 4C).

This first experiment, based on a national declarative survey, calls into question the existence of beliefs surrounding consumer preferences for a particular colour of rose wine. It shows that colour preferences are not unanimous across the different regions of France, despite the existence of a global trend. In fact, consumers in the Provence region, whose wines are traditionally associated with paler colours, stand out from the other two regions (Ile-de-France and Gironde), preferring the intermediary “salmon” shade. Consumers in Gironde and Ile-de-France, on the other hand, prefer the “apricot” shade of Wine D. In contrast, the “lychee” shade of Wine H is typically the least popular amongst all French consumers.

We will demonstrate that this result is reinforced with experiment 2, which uses an incentive-aligned survey, based on the “apricot”, “salmon” and “lychee” shades highlighted in this declarative survey (experiment 1). This second experiment shows, on the one hand, the colour effect demonstrated by the declarative survey, and, on the other hand, confirms the fundamental importance of this visual anchor, which prevails over all intrinsic considerations and also over complementary extrinsic considerations, regardless of whether these are related to the environment or health.

### 3.2. Results from Experiment 2

No impacts from socio-professional, age or gender characteristics were observed during analysis.

A three-way ANOVA (subject, wines and stages) was completed. A significant effect (*p*-value < 0.0001) was observed for the factors “subjects” (F = 13.4; DF = 105) and “wines” (F = 8.6; DF = 3). A post hoc Fisher (LSD) test showed a significant WTP difference between the modified organic wine (average ± CI = 4.2 ± 0.4) or the wine with no added sulphites (average ± CI = 4.4 ± 0.4) and the organic wine (average ± CI = 3.5 ± 0.4) or the conventional wine (average ± CI = 3.7 ± 0.4). The price difference between the two organic wines testifies to this colour effect. The organic wine whose colour had been modified was still preferred, regardless of the stage of the procedure, as is shown in Table 5 below; Table 5 represents the differences in average prices between the two wines. It is important to note that these differences are always in favour of the wine whose colour had been modified.

Nonetheless, the results of the ANOVA show a significant interaction between the factors “stages” and “wines” (F = 2.4; DF = 6, *p*-value = 0.025). In order to further develop use of the data and assess the order of appreciation between the wines at each stage, post hoc Fisher (LSD) tests were completed.

**Figure 2:**
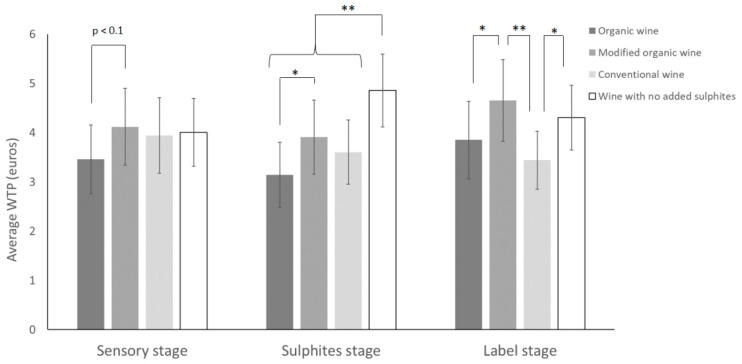
Average WTP (average ± 95% confidence interval) for each wine according to the different stages of the experimental procedure (sensory, sulphites, label). ANOVA, post hoc Fisher (LSD) test, * *p*-value < 0.05; ** *p*-value < 0.01.

At the sensory stage, and therefore in the absence of information on the use of sulphites and organic certification (identified by the AB (organic agriculture) label on wine bottles), the modified organic wine with the “apricot” shade tends, on average (*p*-value < 0.1), to be more highly valued by the consumers than the organic wine with the “lychee” shade (Figure 2). The modified organic wine effectively obtains a 17% higher WTP on average (30% taking the median into account) compared with the organic wine, despite these being the same wine, with only the colour differing between the two wines.

**Figure 3:**
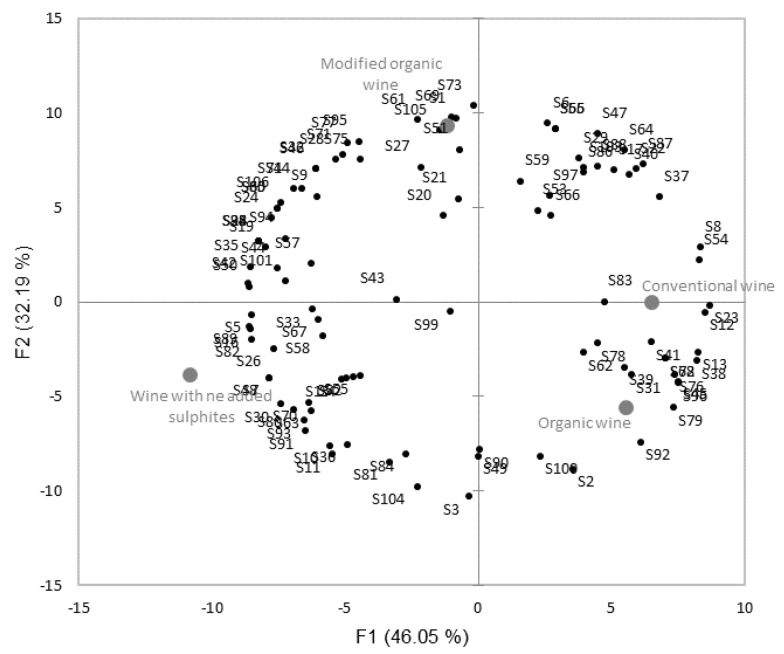
Principal component analysis in the WTP of consumers at the sensory stage. The variables correspond to the judgements of 106 subjects and observations for the four wines presented (78% of variance represented in the first two factors).

If we look in detail at the WTP classifications for each consumer, we can see a range of assessments for the four wines presented. The principal component analysis, Figure 3, illustrates these inter-individual variations in assessments.

Between the sensory stage and the sulphites stage, the WTP for the wine with no added sulphites, with the “apricot” shade, increases on average by over 21%, rising from EUR 4.01 to EUR 4.86 (or 16.25% taking the median into account).

The WTP for this wine is therefore significantly higher (*p*-value < 0.01) than that of all other wines (Figure 2). On the other hand, the inability to use this claim does not significantly reduce WTP (*p*-value > 0.05; analysis between the sensory and sulphites stages not represented in Figure 1). It should be noted that there is still a difference in appreciation between the organic wine and the organic wine whose colour has been modified (*p*-value < 0.05), bearing in mind that the regulatory information on sulphites is the same for both wines and only their colour is different.

At the next stage, consumers were provided with additional information (label stage). Changes were then seen in the order of appreciation for the wines. AB (organic agriculture) certification has the effect of increasing the average WTP for the two organic wines (the organic wine with the “lychee” shade and the modified organic wine with the “apricot” shade) (*p*-value < 0.05 for the modified organic wine and *p*-value < 0.1 for the organic wine; post hoc Fisher test between the sulphites and label stages not represented in Figure 2). At the label stage, the modified wine remains significantly different from the organic wine (*p*-value < 0.05) and is significantly different from the conventional wine (*p*-value < 0.01) (Figure 2).

The inability to benefit from AB (organic agriculture) certification lowers the average WTP of the conventional wine by 4.4% (or 14.3% of the median WTP). The absence of certification, and therefore of the AB (organic agriculture) label, on the wine with no added sulphites lowers the average WTP by almost 11.3% (and the median WTP by 14%). These decreases between the sulphites and label stages are not significant (*p*-value > 0.05), however, in the label stage, we see that the organic wine and the wine with no added sulphites are no longer significantly differentiated by consumers.

These results provide us with information on the effect of a wine’s colour (widely documented in the literature) and the reinforcement of consumer choices with extrinsic information. These overall results do not, however, allow us to draw an absolute conclusion on decisions related to sensory information versus claims or labels. Indeed, we can see that the most popular wines in the sensory stage are those featuring the “no added sulphites” claim or the “organic agriculture” label.

To better understand the weight of intrinsic colour information versus extrinsic sulphites and label information, we decided to test consumer behaviour based on their initial preferences (WTP at the sensory stage). These analyses are primarily focused on two groups of consumers:

- Group 1 was composed of 22 consumers that valued the conventional wine (with the “salmon” shade in the Centre du Rose colour chart) at the sensory stage;
- Group 2 consisted of the consumers that ranked the organic wine (“lychee” shade) last (smallest WTP) at the sensory stage. As the numbers in these groups were small, the data were analysed using a non-parametric Friedman test (paired comparison post hoc Nemenyi test).

Table 6 and Table 7 show that the conventional wine with the “salmon” shade is still the most popular wine among consumers in group 1 in all stages: although the label and health information linked to the use of sulphites have an impact, they do not lead to a reassessment of the classification of wines.

For group 2, which did not like the organic wine at the sensory stage, no significant influence from labels was observed. The AB (organic agriculture) label did not change the preference for the wine initially least appreciated for its organoleptic qualities (Table 6 and Table 8).

This study shows that the consumers in our experiment discriminate against wines based on their colour. Table 5 shows that a slight preference is observed for the “apricot” shade. Indeed, the modified organic wine (“apricot” shade) receives a median 30% higher willingness to pay compared with the organic wine (“lychee” shade on the Centre du Rose colour chart), despite only the colour differing between these two wines. This trend for the “apricot” shade confirms the results of the survey proposed in experiment 1.

For the consumers who ranked the organic wine with the “lychee” shade in last place at the sensory stage, organic certification did not lead to a reassessment of the WTP for this wine. The colour anchor therefore occupies a leading role for the consumer.

We note, however, a certain inter-individual diversity in the sensory judgement of the wines, creating an anchor regardless of the information provided to consumers.

The “no added sulphites” health claim leads to a median 16.25% increase (and average 21.2%) in the WTP for the wine (whose “apricot” shade is very popular) able to make this claim. The WTP for this wine becomes significantly higher than that of all the other wines. On the other hand, the inability to use this claim does not significantly reduce the WTP of the other wines and does not negate the colour effect (difference between the organic wine and the modified organic wine). Once again, organic certification does not change the colour anchor.

Information concerning the “no added sulphites” health claim or AB (organic agriculture) environmental certification does not have a significant impact on the willingness to pay of the consumers who preferred the conventional wine with the “salmon” shade at the sensory stage.

## 4. Discussion

The two experiments presented in this article are in agreement in the sense that the consumers state heterogeneous preferences for the colour of rose wines. Nonetheless, from a geographical perspective, it is possible to observe fairly robust regional preferences. For the Bordeaux region, the “apricot” shade is therefore significantly more popular in our two experiments, at the expense of the “lychee” shade. If we therefore limit ourselves to this type of regional analysis, it is possible to improve the perception of a wine by moving closer to the “preferred” colour of regional consumers: in our case, the median WTP was valued 30% more thanks to a modification to the colour of the rose wine. In comparison, the health claim related to the non-use of added sulphites and organic certification only led to an increase in the WTP of approximately 10%. It is also worth noting that the preferred colour can have a “shielding” effect on the other characteristics of wines. The absence of organic certification or “naturalness” has a significantly less negative impact on a wine whose colour is valued. This result differs from other studies on the popularity of organic wines [54]. It is, however, easily explained by the importance of colour in the characterisation of rose wines and the atypical nature of this market.

Colour thus plays a primordial role in the appreciation of other extrinsic characteristics: it protects wines with the “right” colour and exposes others. We have also verified that organic certification leads to a significant rise in WTP for a wine with an “apricot” shade rather than a “lychee” shade, and, inversely, that the absence of a health claim further lowers the WTP of wines with a less popular colour.

Various authors [55,56] show that the effect of environmental information differs depending on the perceived quality of wines. While this information can, for example, help increase the willingness to pay for wines that are already popular, it will not have an effect on wines that do not satisfy consumers’ organoleptic demands [56]. We have demonstrated that visual information (the most nurtured sense from childhood) has a clearer impact on consumer preferences, which could explain the nuanced results between red and rose wines.

The issue we would most like to raise in this discussion pertains to the explanation of this attachment to colour, which is not simply a matter of decisions made between independent characteristics, and which “positions” the consumer in a specific mode for evaluating wines. Indeed, in our review of the literature, we have tackled the issue of how some authors have been able to demonstrate inferences of quality linked to colour. Although we have not explicitly measured it in our experiment (this could be the subject of future additional studies), this inference has undoubtedly been more or less proven here. The specificity of colours linked to the sensory place rose wines occupy in consumers’ minds therefore takes on a specific emotional character, which must be very explicitly taken into consideration in sensory analyses.

In general, the “emotional” effect of colour and its potential anchor effect has been neither proven nor highlighted in the literature relating to the sensory assessment of wine. Indeed, current knowledge highlights the fact that the emotional charge is more marked for olfaction than for vision [57]. In the case of red wine, studies have shown that emotional responses when tasting a wine (study amongst experts) are more closely linked to olfaction, with visual characteristics having a stronger influence on technical judgements [58]. This observation is found in the literature for products other than wine. In fact, visual perception is directly linked to previously acquired technical knowledge [59]. Additionally, it must not be forgotten that the limbic system (emotions) and olfactory system are closely linked from an anatomical point of view.

A hypothesis could, in the case of rose wines, nonetheless be made that colour may enable consumers to anticipate or derive a strong expectation in terms of the odours, aromas and tastes of wines and therefore the emotions they may experience. For wines with similar characteristics, the aromas of which may be difficult to distinguish, the “olfaction” stage does not have an impact on the associations and expectations generated at the visual stage. The link anchoring colour and emotion could be indirect but stronger for rose wines with a more pronounced colour. This could explain their specificity in the wine market. It should be noted that this hypothesis may be unique to the world of wine. Indeed, while the “apricot” shade of rose wines seems to generally garner a majority preference among the consumers surveyed in the Bordeaux region, a similar survey on pens, for example, would almost certainly have led to different results. Different behaviours should be expected for food and drink and non-food and drink products, notably because in the former, colour plays a role in the anticipation of flavours and aromas.

## 5. Conclusions

We conclude this article with observations on the atypical nature of the rose wine market and on possible avenues for further research related to the emotional role colour plays in wine tasting and its possible specificity in the world of food and drink products. It is important to evaluate trade-offs between intrinsic information and extrinsic information to better understand consumer behavior and the current market. These choices vary depending on the type of wine and its quality level.

## Figures and Tables

**Table 1:** Structure of the panel by geographical area.

		Geographical Area		
		Ile-de-France	Bouches-du-Rhone	Gironde
Age	20–34 years	33%	27%	40%
	35–54 years	34%	32%	34%
	55 years or more	34%	39%	28%
Gender	Male	30%	37%	34%
	Female	36%	31%	33%
Frequency of wine consumption	Daily or almost every day	31%	35%	35%
	1–2 times a week	35%	28%	37%
	Less than once a week	33%	38%	29%
Frequency of rose wine consumption	More than 10 times a year	28%	39%	33%
	4–10 times a year	39%	25%	36%
	3 times a year or less	55%	20%	25%

**Table 2:** Technical and analytical characteristics (FOSS^®^ analysis) of wines selected.

	Conventional Wine	Organic Wine	Wine with No Added Sulphites
Grape varieties	Cabernet Franc, Cabernet Sauvignon, Merlot	Cabernet Franc, Merlot	Merlot
Added sulphite content	Max 150 mg/L	Max 50 mg/L	/
Shade (rose wine colour chart)	Salmon	Lychee	Apricot
Sale price (ex-works)	EUR 5.90/bottle	EUR 6.85/bottle	EUR 6.90/bottle
Alcohol by volume	12.86	13.06	12.76
Total acidity in H_2_SO	3.33	3.61	2.53
(sugar) (g/L)	0.4	1.1	0.9
pH	3.4	3.4	3.4
OD280	7.7	8.1	5.1
OD420	0.137	0.14	0.169
OD520	0.382	0.391	0.42
OD620	0.078	0.076	0.08

**Table 3:** Results of the survey on the assessment of the colours of rose wines.

Wine	Wine A	Wine B	Wine C	Wine D	Wine E	Wine F	Wine G	Wine H
“Parfaite” (perfect)	5.5%	7.5%	7%	21%	15%	7%	6%	5%
“Tres bien” (very good)	8%	18%	17%	24%	26%	23%	16%	11%
“Assez bien” (fairly good)	14%	21%	21%	26%	22%	20%	22%	14%
“Moyenne” (average)	16%	18.5%	21%	15%	19%	18%	20%	18%
“Pas terrible” (not great)	27%	23%	23%	10%	11%	18%	21%	26%
“A rejeter” (rejected)	29.5%	12%	11%	4%	7%	14%	15%	26%
Total respondents	450	450	450	450	450	450	450	450
Majority statements	“Pas terrible” (not great)	Average	Average	“Assez bien” (fairly good)	“Assez bien” (fairly good)	“Assez bien” (fairly good)	Average	“Pas terrible” (not great)
% obtained by the majority statement	71%	65%	66%	71%	63%	50%	64%	74%

**Table 4:** Results for respondent preferences for the colour of rose wines using the Balinski and Laraki method (2011) for the Ile-de-France (**A**), Gironde (**B**) and Bouches-du-Rhone (**C**) regions.

**A**								
**Ile-de-France**								
	**Bottle A**	**Bottle B**	**Bottle C**	**Bottle D**	**Bottle E**	**Bottle F**	**Bottle G**	**Bottle H**
“Parfaite” (perfect)	4%	5%	4.5%	26.5%	14%	3%	3%	2.5%
“Tres bien” (very good)	9%	16%	18%	21%	21%	17.5%	13%	10%
“Assez bien” (fairly good)	14%	25.5%	24%	24.5%	25%	21%	25%	12%
Average	19%	19.5%	21%	19%	21%	21%	20.5%	18%
“Pas terrible” (not great)	29%	21%	20%	5%	13%	23.5%	22%	31%
“A rejeter” (rejected)	25%	13%	12.5%	4%	6%	14%	16.5%	26.5%
Total	150	150	150	150	150	150	150	150
Majority statements	“Pas terrible” (not great)	Average	Average	“Assez bien” (fairly good)	“Assez bien” (fairly good)	Average	Average	“Pas terrible” (not great)
% obtained by the majority statement	75%	66%	67%	72%	60%	63%	61%	73%
**B**								
**Gironde**								
	**Bottle A**	**Bottle B**	**Bottle C**	**Bottle D**	**Bottle E**	**Bottle F**	**Bottle G**	**Bottle H**
“Parfaite” (perfect)	10%	14%	10%	17%	10%	4%	1%	-
“Tres bien” (very good)	11%	26%	21%	27%	21%	12.5%	10%	7%
“Assez bien” (fairly good)	19%	22%	21%	27%	20%	23%	19%	15%
Average	18%	19%	23%	16%	21%	21%	22%	18%
“Pas terrible” (not great)	25%	15%	18.5%	9%	17%	19%	29%	27%
“A rejeter” (rejected)	17%	4%	6.5%	4%	11%	20.5%	19%	33%
Total	150	150	150	150	150	150	150	150
Majority statements	Average	“Assez bien” (fairly good)	“Assez bien” (fairly good)	“Assez bien” (fairly good)	“Assez bien” (fairly good)	Average	Average	“Pas terrible” (not great)
% obtained by the majority statement	58%	62%	52%	71%	51%	60%	52%	67%
**C**								
**Bouches-du-Rhone**								
	**Bottle A**	**Bottle B**	**Bottle C**	**Bottle D**	**Bottle E**	**Bottle F**	**Bottle G**	**Bottle H**
“Parfaite” (perfect)	2%	3%	6%	20%	20%	14%	13%	11%
“Tres bien” (very good)	5%	13%	11%	25%	35%	38.5%	25%	17%
“Assez bien” (fairly good)	9%	15%	18%	25%	22%	18%	23%	17%
Average	11%	17%	19.5%	10%	14%	12%	17%	18%
“Pas terrible” (not great)	27.5%	31%	31.5%	16%	3%	11%	13%	20%
“A rejeter” (rejected)	45.5%	21%	14%	4%	6%	6,5%	9%	17%
Total	150	150	150	150	150	150	150	150
Majority statements	“Pas terrible” (not great)	“Pas terrible” (not great)	Average	“Assez bien” (fairly good)	“Tres bien” (very good)	“Tres bien” (very good)	“Assez bien” (fairly good)	Average
% obtained by the majority statement	55%	79%	55%	70%	55%	53%	62%	63%

**Table 5:** Average willingness to pay (WTP) (± standard error) for the two wines featuring the organic certification label for each stage in the procedure (WTP shown in euros). Delta = the difference in average prices between the modified organic wine and the organic wine.

	Organic Wine	Modified Organic Wine	WTP Delta (%)
WTP sensory stage	EUR 3.46 ± 0.39	EUR 4.12 ± 0.39	+17%
WTP sulphite regulations stage	EUR 3.14 ± 0.35	EUR 3.91 ± 0.38	+24.5%
WTP label information stage	EUR 3.85 ± 0.4	EUR 4.65 ± 0.42	+21%

**Table 6:** Sum of WTP ranks obtained for the group of consumers who liked the conventional wine (group 1—22 consumers).

		Sum of Ranks		
		WTP Sensory Stage	WTP Sulphites Stage	WTP Label Stage
Organic wine	“Lychee” shade	53	52	58
Modified organic wine	“Apricot” shade	42.5	40.5	47.5
Conventional wine	“Salmon” shade	88	81.5	76.5
Wine with no added sulphites	“Apricot” shade	36.5	46	38

**Table 7:** Results of Friedman tests for comparison of WTP ranks for each wine and at each stage of the experiment for consumer group 1 (22 consumers). Friedman test, post hoc Nemenyi test; NS: not significant; ** *p* > 0.01.

	WTP Sensory Stage	WTP Sulphites Stage	WTP Label Stage
No added sulphites vs. modified organic	NS	NS	NS
Organic vs. modified organic	NS	NS	NS
Conv. vs. modified organic	**	**	**
Conv. vs. no added sulphites	**	**	**
Conv. vs. organic	**	**	NS
Organic vs. no added sulphites	NS	NS	NS

**Table 8:** Results of Friedman tests for comparison of WTP ranks for each wine and at each stage of the experiment for consumer group 2 (21 consumers). Friedman test, post hoc Nemenyi test; NS: not significant; * *p* > 0.05; ** *p* > 0.01.

	WTP Sensory Stage	WTP Sulphites Stage	WTP Label Stage
No added sulphites vs. modified organic	NS	NS	NS
Organic vs. modified organic	NS	NS	NS
Conv. vs. modified organic	NS	NS	NS
Conv. vs. no added sulphites	**	**	**
Conv. vs. organic	**	**	**
Organic vs. no added sulphites	**	**	*
